# American Medical Association

**Published:** 1873-06

**Authors:** 


### *American Medical Association.*

[As our Special Correspondent, detailed for the occasion, has failed to report on time for the present No., we borrow *verbatim et literatim*, without endorsement, the report of *The Clinic*.—Ed.]

St. Louis, Tuesday, May 6, 1873.

The National American Medical Association assembled this morning at 11 o'clock at Masonic Hall, Corner of Seventh and Market Streets.

The meeting was called to order by the ex-President of the Association, Dr. D. W. Yandell, of Louisville, who introduced Rev. Dr. Nicholls, who opened the session with prayer. Dr. Nicholls concluded with the Lord's prayer.

The chairman then introduced to the convention Dr. John S. Moore, one of the veteran physicians of St. Louis, who had been chosen to deliver the welcoming address.

As usual upon such occasions, the Dr. exploded at once into a grandiloquent burst of oratory. He congratulated his audience, some 300 in number, on the fact that the far-famed "Star of Empire" was just at that moment so far west and so high in the zenith as to be able to shine upon their deliberations. He spoke of the "Father of Waters" which flowed at their feet, of the fertility of the soil of the State of Missouri, of the city of St. Louis as the "Metropolis of the West," [what Western city is not?] containing [everything must be grand in proportion] no less than 450,000 souls. Having wrought up his audience to the proper pitch with these new and interesting facts, in a medical point of view, he exhibited to them that these things were mere trifles when compared with the vast amount of ores which lay imbedded beneath their feet, particularly of lead and iron. "Why, gentlemen," said he, glowing over the thought like a heated furnace, "not a hundred miles below this city, on the Iron Mountain Railroad, we not only have an iron mountain but iron mountains—sufficiently abundant to supply the world with this important metal for thousands of years—mountains made up of iron ore so pure as to be manufactured into horse shoes and horse-shoe nails on the blacksmith's anvil," and then, half-deprecatingly, half-jestingly, "I will not vouch, however, for horse-shoe nails." (Appreciative applause).

In explanation of the fact that St. Louis had no medical edifices or hospitals or museums and such like paraphernalia, to speak of, the speaker hastens to explain that "the people of this city did not come here, particularly, to found and foster scientific and benevolent institutions. What do you suppose, sirs, they came here for? To make money—to worship at the altar of mammon, and not the altar of science. This city, consequently, viewed from a scientific stand-point, does not compare as favorably as we might desire with some of the other cities of this country. * * * * I am glad, however, gentlemen, to be able to say that it is contemplated soon to construct a general hospital, under the auspices of both the State and the city, which will doubtless be ample for all our wants.

"In conclusion, notwithstanding we cannot point you to magnificent medical edifices, extensive museums, and capacious hospitals, we hope to make amends, in part at least, for all these short-comings, by the cordiality with which we receive you."

The retiring President of the Association then introduced his successor, Dr. Thomas M. Logan, the newly-elected President, who came forward and took his seat.

Dr. J. B. Johnson, Chairman of the Committee of Arrangements, made a few remarks in furtherance of the welcome extended by Dr. Moore. He thought that his predecessor had almost exhausted the subject, but he could not refrain from extending the congratulations of the committee which he represented to the guests of the St. Louis medical fraternity, but wished to add their hearty welcome to the one already extended.

The committee recommend that the general meeting be held each morning in the main hall, from 9 A. M. to 1:30 P. M.

Further, that the committees, known as "sections," meet as follows: Section on Surgery, in main hall, and other sections on the third floor. Section on Materia Medica and Chemistry, in the exposition room. Section on Psychology and Medical Jurisprudence, in the "entered apprentices' room." Sections on the practice of medicine and obstetrics, in the master masons' room. Exposition of drugs, chemicals, instruments, chemical apparatus, and surgical appliances, in the banquet hall.

Dr. Logan now came forward and delivered himself of

*The Presidential Address.*—Every thoughtful medical man who attended this late meeting of the American Medical Association, must have felt humiliated when he heard the ranting twaddle uttered by the newly installed President, Dr. Thos. M. Logan, of California. This is not the first time that the association and the profession of the country have been chagrined by the wretched spectacle of an incompetent and pretentious ignoramus roaring his senseless but sounding phrases in the ears of the public. It was said by Napoleon on his retreat from Russia, "there is but a step from the sublime to the ridiculous." The American Medical Association, if ever sublime has certainly descended to the ridiculous. This stage appears to have been inaugurated by your very respectable fellow citizen—Dr. George Mendenhall, whose address was considered remarkable, only for its puerilities, and whose quality as presiding officer was fitly characterized by an Eastern Medical Journal, as "palsied inefficiency." Dr. Mendenhall did not attempt eloquence. He was wisely ambitious to appear in the *role* of the old family horse, who never kicks up in the shafts nor runs away. He has been followed by two eloquent presidents—Drs. Yandell and Logan—race horses, low down in the "forties." These orators burst on the Association, breathing flames and smoke—terms which, we doubt not, will be considered by themselves to appropriately designate their rhetorical pyrotechnics. Space will not admit, nor is life long enough, to review the wonderful performance of Yandell, but our readers are entitled to some specimens from the fast, furious and formidable oration of Logan: Listen O! awe-struck reader of THE CLINIC to this exordium:

"GENTLEMEN: Just two years ago there was witnessed a spectacle well worthy our contemplation! It was full of significance, and stands forth, unparalleled, in the history of our divine art, from its earliest annals down to the present moment.

"Along the Atlantic slope of this vast continent—throughout the length and breadth of the land from Maine to Mexico—were seen, gathering together, one hundred and twenty-one living, aspiring intelligences, moved by one thought, nerved by one impulse, animated by one hope—the good of humanity!

"Abandoning, for the nonce, the peaceful pursuits of their chosen vocation, relinquishing its rewards, and exposing themselves to all the hazards incident upon velocity of locomotion, westward they steered their beneficent course, borne along the iron pathway cleaved across the continent!

"Annulling the opposing conditions of time and space, over three thousand miles they went—'skimming over the valleys, thundering across the rivers, and panting up the sides or piercing through the hearts of the mountains.' Science having made subservient to their bidding those dynamic agencies, more potent than the Genii of Arabian fable, they accomplished in seven days the travel that once consumed more than as many months: and thus they reached the city of the Golden Gate—the Mecca of their pilgrimage."

This tremendous burst of eloquence was roared out to commemorate the fact that one hundred and twenty delegates from the States east of the Rocky mountains attended the meeting of the Association at San Francisco. Further on the great orator characterizes this bit of travel as "a spectacle of moral grandeur never before witnessed in the history of the Association—never in the annals of medicine." The actors in this wonderful performance appear to agree with Dr. Logan in their estimate of its importance, for they have organized themselves into a society under the title of the "Rocky Mountain Society" and have elected Dr. Washington L. Atlee, President. We have not been favored with the "eloquent" address of Dr. Atlee, but we can affirm in advance that it is as tremendous as the flowery, poetical, sentimental, pious,—and we must say, unintelligible—address of Parvin, President of the Medical Journal Association, a society, also, whose existence is a "spectacle of moral grandeur," etc. The American Association, O! unsophisticated reader of THE CLINIC, contains a vast number of eloquent gentlemen; but one President is elected annually; hence the infrequent opportunities for rhetorical display, and hence, also, the organization of the Medical Journal Association, of the Rocky Mountain Association, and possibly, other spectacles of moral grandeur. Let them all do their best: we believe that none of them will ever attain to the moral grandeur achieved by the eloquent Logan.

After the exordium above given, Dr. Logan expatiates on the great influence of the Association in the same stately manner, and with great smoke and flame, as follows:

"Nor is the influence yet abated. Like yon mighty river, which sweeps with ever-living, ever-moving waters along the wharves and by the busy marts of this Empire City of the West, carrying rich deposits of fertility and plenty from State to State, in its annual overflowings, to bless the dwellers upon its shores and throughout the vast regions of its lengthened course, so we are here to-day, rejoicing in the strength of our numbers, to scatter far and wide, along the pathway of humanity, the benign influences and free-will offerings of our collective counsel and experience."

He gratifies the assembled medical wisdom by this tribute to the influence of the Association:

"But, gentlemen, if the estimate I have rendered of what our Association have done be at all true—if it has made better physicians of us and raised the dignity of the profession—if it be at all true that the infusion of clear and inductive thinking, and the importation of scientific method and scholastic art, have done so much to advance medicine towards that exalted station among its cognate sciences to which it is so justly entitled—then so much the weighter are our present responsibilities; so much the louder is the call upon us to sustain our lofty character and position, by increasing the expansive circle of our usefulness, and by extending the range of our scientific resources."

Dr. Logan takes issue with Yandell on the matter of medical education, in the same eloquent strain. Yandell had in the previous meeting roared over the "rugged utility" aspect of medical education, and gave the *coup-de-grace* to scholastic attainments. Logan arises to a noble indignation when he reaches this point, and delivers himself in the following tremendous style:

"In vain may cry of 'Reform! reform!' be rung with its many changes round the circle of our schools, from Maine to Louisiana, and be re-echoed from our colleges in California and Oregon, so long as it is proclaimed in the high places of the profession, that the exigencies of the times and the requirements of humanity exact such a constant supply of medical force, as will hardly permit the acquisition of any greater degree of knowledge and attainments than such as will enable the new-fledged graduate to turn them promptly to clinical purposes. With an apparently reckless inconsiderateness of what might entail a waste of professional intellect, that may possibly be equivalent to the daily loss of threescore and ten years of progress, it has been deliberately argued, in the presence of the assembled Association, that profundity of learning is not essential for the discharge of the physician's function, and that practically the more the sphere of his scientific resources is expanded, so much the less ability does he seem to exhibit in the use of therapeutics. Germany has been instanced to substantiate this hypothesis—Germany, where the crowning glory of modern medicine is found, not only in its minute and exact knowledge of general, special and comparative anatomy and physiology—not only in those peculiarities of the therapeutics of to-day, that one of the freshest and most advanced thinkers of the age has termed *Restorative Medicine*, in contradistinction to destructive and depressing medication—but rather in 'that purer jewel of her crown,' unblemished by the slightest taint of selfishness—*Preventive Medicine ;* Germany, where cellular pathology is sweeping into oblivion a long catalogue of torturing and depressing agents, and where an amount of research in the natural history of diseases, while putting to shame our own shortcomings, is urging us, in common with the prosecutors of our science everywhere, to more determined efforts in this respect—there, in that 'vater-land,' its therapeutics have been signalized as 'something hardly better than nihilism,' and the practice of physic not much more than a 'meditation on death.'"

Having vindicated the claims of scientific medicine, Dr. Logan in the same grand style falls upon the subject of a National Sanitary Bureau, which he warmly advocates. He next proposes that the "work for the future" of the Association is to "educate the people." To this end, he suggests that they "throw away all puerile notions about the dignity of our calling, and approach the people through the only channels by which they can be reached—the newspaper and the lecture room."

Dr. Logan concludes his address in the following peroration, which as a final burst of 2:40 eloquence is worthy of being studied :

"But, gentlemen, whatever course you may think proper to pursue, I am sure that your objects will be the advancement of science, the good of humanity, and the honor and glory of our beloved profession, which for a continuous period of more than two thousand years has numbered among its votaries many of the wisest and most beneficent of the long roll of sages and philanthropists. I feel, therefore, that I cannot better conclude than by bidding you, in this connection, hearken to the utterances of Missouri's honored son, *Clarum et venerabile nomen*, pronounced eighteen years ago before this Association, and now echoed back, from his sepulchral couch, in the Capital of France :

"'On the eve of the battle of the Pyramids, Napoleon exclaimed—'Soldiers ! from the height of yon monuments forty centuries look down upon you.' Gentlemen, from the heights of past ages countless worthies of our God-like profession point and beckon to a goal more elevated than ever attracted legislators and conquerors, Solons and Cæsars !'"

Is it not plain that there is but a step between the sublime and the ridiculous ?

*Entertainments.*—The local committee reported the following arrangements for the pleasure of visiting members :

This evening there will be a *soiree musicale* at Mercantile Library Hall, commencing at 8 o'clock. A band has been engaged, and arrangements made for refreshments. The wives and daughters of the physicians are expected to be present. To-morrow evening there will be held a *levee* at the residence of Colonel J. L. D. Morrison, corner of Locust street and Leffingwell avenue. On Thursday evening, Dr. J. J. Woodward, U. S. A., of Washington, will, by special request of the Committee of Arrangements, deliver the Toner Lecture at Masonic Hall. The announcement was received with great applause. On Friday afternoon the visitors will view Lafayette Park, Tower Grove, and Shaw's Garden. Carriages to leave the hall at 2 o'clock.

*Special Orders.*—A letter from Dr. Woodward, U. S. A., on the organization of the medical corps of the army, was made the special order for to-morrow. Dr. Moore, chairman of the Committee on Prize Essays, notified the committee to meet at his office at 3 o'clock to-morrow afternoon. An invitation was received from the St. Louis Mutual Life Insurance Company to visit their magnificent building. Chauncey I. Filley, postmaster of St. Louis, placed an elegant mail box in the hall, and notified members that every facility would be afforded visiting delegates to receive and dispatch their letters without delay. (Applause.)

*Meeting of Sections.*—In the afternoon the Convention sat by sections, devoted to the consideration of the various branches of medical practice, and proceeded to discussions and reading of essays on topics pertinent to the several departments below named.

*Surgery.*—The meeting was convened at 3 o'clock. In the absence of the president, Dr. Warren, Dr. Lankford was elected to the chair; Dr. W. F. Peck, of Iowa, acted as secretary.

Dr. Andrews invited the attention of the profession to the use of drainage tubes in cases of pyæmia, in connection with abscesses in the chest.

Dr. Gouley, of New York, complimented the use of Chassignac's drainage tube, preferring it to the pneumatic aspirator.

The subject was further discussed by Dr. Hughes, who referred to a novel method pursued by Dr. Stone, of New Orleans, in treating abscesses in the chest—taking out a piece of rib, and thereby furnishing an opportunity for permanent drainage; and remarks were also made by Dr. Jones, of Chicago.

The subject of consecutive dislocation of the elbow was brought up by Dr. Moore, of New York, and discussed by Dr. Allen, of Louisiana, and Dr. Andrews.

Dr. Gouley, of New York, introduced a new instrument employed in arresting hemorrhage in cases where the peritoneum is divided, in cases of stone in the bladder and stricture of the urethra.

*Practice of Medicine and Obstetrics.*—The section met at 3 o'clock in the grand masters' room, Dr. O'Donnell, of Baltimore, in the chair, and Dr. W. Clark secretary pro tem.

The subject of treatment of puerperal convulsions was first discussed. Dr. Hasham, of Delaware, advocated the old remedy, the lancet, expressing the opinion that chloral and its substitutes were quite inefficient. Dr. Catlin, of Connecticut, thought the lancet effective in apoplectic cases, but chloral, stramonium, bromide, etc., in anæmic cases. Dr. Helm reported a case where a patient lost 252 ounces of blood, and recovered. Dr. Hasham maintained that convulsions caused the death of the child. Dr. Montgomery, of St. Louis, advocated the lancet in old and apoplectic cases. Dr. Bean, of New York, and Dr. Gaines, of Alabama, participated in the discussion.

Dr. Seguin, of New York, then read an interesting essay on "Education of the Medical Senses," which was referred to the Committee on Publication.

Dr. Smith, of Iowa, made remarks on position in labor, relating a case of lateral oblique corrected by gravity, and was followed by Dr. Maughs, of St. Louis, and one or two others.

*Psychology and Medical Jurisprudence.*—The hurry connected with arrival, and the more practical interest attaching to the work of other sections, injuriously affected the attendance on this section, and only about a dozen delegates assembled in the entered apprentice room, where the sessions are to be held. No organization was effected, and the meeting adjourned to next day. Several interesting papers on the medico-legal question of insanity and other kindred topics of great public interest are said to be forthcoming.

*Materia Medica and Chemistry.*—In a room opposite that occupied by the section on obstetrics and practice of medicine, is an interesting exposition of materia medica and the chemicals, apparatus, etc., used in medical practice. The room was only partly filled, but other articles will probably be exhibited in a day or two.

SECOND DAY.

The Convention was called to order this morning at 10 o'clock, Dr. Logan, of California, in the chair.

*The Mercantile Library.*—Dr. Atkinson, of Philadelphia, Secretary of the Association, read a communication from the secretary of the Mercantile Library, inviting them to visit the Library rooms during their stay in St. Louis. The invitation was accepted.

*Nominating Committee.*—The President directed that each State select a delegate to represent them on this committee. The Secretary called the roll of the committee so constituted.

*Invitation from Detroit.*—The Secretary read invitations from the medical societies of Detroit and the State of Michigan, asking the American Medical Convention to select Detroit as its next place for annual meeting. Referred to the Nominating Committee, which retired for deliberation.

*Meetings of Sections.*—A Special Committee to report a plan for the better arrangement of "sections," through its chairman, Dr. Howard, of Baltimore, made a report, with a view to expedite business.

The committee suggested that all lengthy papers be referred to a sub-committee, to determine as to the advisability of bringing them before the sections. Each section to have a sub-committee for this especial purpose.

Effective action was urged upon the important subject of medical instruction. That all questions of a personal character, complaints and on credentials, be referred to the Committee on Ethics without discussion. That the Committee on Ethics be constituted of not less than nine members, to be selected by the Nominating Committee and to serve for three years. The following arrangement for sections was suggested in place of that now existing:

1. Practical medicine, materia medica and physiology.
2. Obstetrics, and diseases of women and children.
3. Surgery and anatomy.
4. Medical jurisprudence, chemistry and psychology.
5. State medicine and public hygiene.

*The Annual Storm Brews.*—Dr. Bronson, of Massachusetts, desired that the report of the Special Committee in reference to arrangement of sections be completed. It was incomplete.

Dr. Davis moved that the report be received, and the recommendations accompanying it be adopted, except such as referred to the Code of Ethics.

Dr. Porter rose to a question of privilege. He said the Code of Ethics needed no change; we need improvement. The interruption of time by voluminous reports worked mischief to the organization. Who reads them? I conceive we want improvement in this behalf. We must not make a mountain out of a mole hill. (Gentle stamping upon the floor, which finally culminated in thunders of applause (?), so that the speaker could not proceed.)

The President suggested that the speaker had exhausted the alloted time for discussion.

Dr. Davis, of Chicago, introduced an amendment to the by-laws that a council, consisting of twenty-one members, shall take cognizance and decide all questions of an ethical or judicial character. This committee to be appointed by the Nominating Committee. After prolonged debate this amendment was voted down, when the report of the Special Committee was adopted.

From Dr. Gross, of Pennsylvania, an interesting letter was read. He stated therein his deep regret that he was unable to attend the present meeting of the Association, and trusted that the meeting would be an uncommonly harmonious one, and that no outside or evil influences might be permitted to mar its deliberations. In conclusion, he proposed certain changes in the laws of the Association, so as to make it more of a scientific and less of a declamatory body than it has heretofore been.

*The Medical Corps of the Army.*—Dr. Woodward, Assistant Surgeon U. S. A., and of the Surgeon General's Department, read a communication to the American Medical Association, signed by a large number of surgeons and assistant surgeons of the U. S. Army, the argument of which shows that the Medical Staff of the United States Army has not been placed on an equal footing with the other staff corps of the army as regards rank, that they have not had the same consideration shown them in this respect as has been accorded to the navy, and that the record of services of this meritorious body of officers entitles them to the same advantages that have been granted to others.

Dr. Woodward said: Now, Mr. President, I had nothing to do with drawing up this letter, but I indorse it fully, as does also the Surgeon General. It is understood that by the provisions of the Army regulations we of the Army are debarred from making such an appeal, but we can aid the suggestion verbally, by appealing to you, gentlemen of the Association. Any army officer, except a Surgeon, can be retired in old age with a competent pay and the rank of Colonel, while a medical man can only have the rank of Major after twenty years of service, and may ask for a Lieutenant Colonelcy at thirty years. A Lieutenant Colonelcy is all we ask. I was an Assistant Surgeon twelve years ago, and hold the same rank yet. I have filled my contract with the Government, but it has not fully complied with its obligations. We have come up to the Convention for help. Will you help us? (Loud applause.) We only ask what was done for our brethren of the Naval Medical Corps, and nothing more.

Dr. Keller, of Louisville, was in favor of asking the Government for the aid required for the Army doctors.

Dr. Yandell concurred, but there was one word he wanted omitted, and that was the reference to "rebel" prisons. He wanted that to read "Confederate" prisons.

*Foreign Representatives.*—The Secretary read the following appointments made by the President to represent the American Medical Association to the British Medical Association. Drs. F. G. Smith, C. Wister, J. C. Cohen, of Philadelphia; Dr. E. Warren, of Baltimore; Dr. C. L. Ives, of New Haven; Dr. Edward Montgomery, of St. Louis; and Drs. F. Barker, E. Seguin, and J. C. Hutchison, of New York.

The Secretary stated that commissions for these gentlemen would be made out at once.

*Report of Nominating Committee.*—Dr. Johnson, of St. Louis, Chairman of the Nominating Committee, made the following partial report:

Your committee suggest the following gentlemen for the various offices named:

President—Dr. J. M. Toner, District of Columbia. First Vice-President—Dr. W. Y. Gadbury, of Mississippi. Second Vice-President, Dr. J. M. Keller, of Kentucky. Third Vice-President—Dr. W. C. Husted, of Missouri. Fourth Vice-President—Dr.

L. D. Warner, of Massachusetts. Treasurer—Dr. Caspar Wistar, of Philadelphia. Librarian—Dr. Wm. Lee. Committee on Libraries—Dr. Johnson Elliott. Secretary—Dr. Theodore A. McGraw. Committee of Arrangements—Dr. Brodie, Chairman; Drs. Jas. A. Brown, Morse Stewart, J. F. Noyes, E. W. Jenks, Henry F. Lyster, D. O. Farrard, Eugene Smith, all of Detroit. Committee on Prize Essays—Drs. J. K. Johnson, A. Sager, H. Hitchcock, of Detroit; E. Andrews, Ill.; E. S. Gaillard, Ky. Committee on Publication—Drs. F. G. Smith, W. B. Atkinson, D. Morrey Chester, of Penn.; Wm. Lee, District of Columbia; Caspar Wistar, Penn.; H. F. Askew, Delaware; Alfred Stille, Penn.

Detroit was named for the next annual meeting of the Association.

The report was adopted.

*Report on Medical Education.*—Dr. Carson, of Ohio, read a very interesting and important report on medical education. He set forth the general (?) dissatisfaction among the profession on the subject. He defined two classes of influences reacting on medical education—the external and the internal—of all of which it may be said that they are the products of growth under certain definite and adherent impulses, with power of adaptive modification. This Association can exert great influence by increasing its efficiency, as a tribunal before which subjects may be brought for the exercise of that most potent influence, *discussion*, and not for that impotent purpose of expecting to exercise legislative functions towards the teaching bodies.

The report was referred to the Committee on Publication.

*Report on Medical Literature.*—Dr. Yandell, Jr., of Louisville, read the report on "Medical Literature." The Association at its last annual meeting requested Dr. Flint to prepare the report, but at the latter's request Dr. Yandell assumed the duties.

He referred to the fact that for years the profession in America had depended entirely on the medical literature of England and the European continent. Now the medical journals published in America are ahead of the combined medical publications of England and France. This remarkable growth must be gratifying to the pride of every American physician. Going back only a quarter of a century and our country was nearly destitute of original works on medicine, almost entirely dependent upon European surgeons and physicians for instruction—now we are independent of the world; our own authors can supply all text books required by the medical student and the practitioner, and for clearness and fullness of information on all practical points, they compare most favorably with the best writings of our brethren abroad.

Dr. Yandell then discussed at length the medical journalism of

America. Over forty medical journals are now published in the United States.

The report was referred to the Committee on Publication.

*Prize Essays.*—The Committee on Prize Essays, of which Dr. John S. Moore, of St. Louis, is chairman, reported that only one such production had been received by the committee, which was not adjudged worthy of any of the prizes offered.

*Treasurer's Report.*—A report was read from the Treasurer of the Association, setting forth that there was a balance in the Treasury of only $450—owing to the heavy expense attending the publication of the proceedings of the last annual meeting.

*The Centennial Celebration.*—Dr. Frederick Horner, Jr., U. S. Navy, offered a resolution that the American Medical Association appoint a committee of one member from each of the original thirteen States of the Union, to report to the Centennial Celebration on the medical, surgical and biographical literature of the period of 1776.

As a tribute to Joseph Warren, Benjamin Rush, Arthur Lee, General Hugh Mercer, and other noble and patriotic physicians who aided to secure American Independence, the resolution was adopted.

*Medicine and War.*—Dr. Peck, of Iowa, introduced the following preamble and resolutions :

In view of the fact that the reports of the Surgeon General of the U. S. Army, as exhibited in volumes one and two of the first part of the Medical and Surgical History of the War of the Rebellion, have received a too limited circulation by reason of an insufficient issue of the same by Congress ; therefore,

*Resolved*, That the President and Secretary of this Association be directed to petition Congress at the next session in behalf of the profession, asking that the addition recently issued be reproduced in sufficient number to permit of general distribution to the members of the profession throughout the country.

*Resolved*, That the thanks of this Association are due and are hereby tendered Congress for aiding thus far in developing and presenting to the profession the reports of the Surgeon General, as herein specified.

*Resolved*, That the thanks of this Association are hereby tendered the officers of the United States Army who have by sacrifice and labor been instrumental in placing before the profession the valuable information contained in volumes one and two of the first part of the Medical and Surgical History of the War of the Rebellion.

Carried unanimously.

*Reception To-Night.*—This evening, at eight o'clock, Col. and Mrs. J. L. D. Morrison will receive the delegates of the American Medical Association at their elegant mansion, corner of Locust street and Leffingwell avenue.

*Medical Statistics.*—Dr. Toner submitted some valuable statistics concerning medical societies and institutions in the United States.

There are 405 of the former, and 174 medical hospitals, besides 82 public hospitals, treating 144,750 patients annually.

*Meeting of the Sections.*—The different sections into which the convention is subdivided, to consider separately the different branches of medical science, meet at 3 o'clock, and were all well attended.

*Surgery.*—The meeting was presided over by Dr. Andrews, of Chicago, Dr. Peck, of Iowa, acting as secretary.

Dr. Lathrop, of Lyons, Iowa, read a paper on the treatment of bunions, referring to new apparatus and improved modes of treatment.

The discussion was confined almost exclusively to this subject, and comprised remarks by Drs. Moore, of Rochester; Brady, of Princeton; Miles, of Indiana; Lucas, of Illinois; Chambers, of Illinois; Bronson, of Massachusetts, and others.

*Practice of Medicine and Obstetrics.*—The chair was occupied by Dr. O'Donnell, of Baltimore, Dr. E. W. Gray, of Bloomington, Ill., acting as secretary.

In response to the president's call, no papers were presented, and on motion of Dr. Gray a request was made that all papers belonging to the section be placed in the hands of its officers.

Dr. Todd, of Kansas City, addressed the section on the subject of physiological anomalies in the configuration of the bony and soft walls of the pelvis as a cause of retardation in labor.

The points of the paper were reviewed by Dr. Hollister, of Illinois, who also made some remarks on secondary hemorrhage after delivery.

The hour having arrived, Dr. Drisdell, of Philadelphia, read his paper on the microscopical appearance of ovarian tissue, in the course of which he announced the discovery of a new cell which he denominated the ovarian granular cell. The paper was referred to the Committee on Publication.

Dr. Howard presented a memorial paper by an absent member, on medical education, which was referred to a special sub-committee consisting of Drs. Howard, Pallen, and Todd.

General remarks of a practical character were made by Dr. Garric, of New York, and others, continuing until nearly five o'clock.

*Psychology and Medical Jurisprudence.*—This section met in the session hall of the Convention, Dr. Comegys, of Cincinnati, in the chair; Dr. Hazard, of St. Louis, secretary.

On invitation of the section, Dr. Hughes, of St. Louis, read a paper entitled "Brain Diseased and Mind Deranged," advocating the peculiar physiological views he has set forth in the medical society. The paper was listened to with close attention, and was apparently received with considerable favor.

Dr. J. B. Hough, Professor of Miami Valley Institute, read an interesting and strongly original essay on "New Methods of Experimentation in the Problem of Spontaneous Generation."

THIRD DAY.

The delegates re-assembled this morning at half-past nine, in the main hall of the Masonic building. The attendance was fully as large as on the preceding day. Dr. Logan in the chair, with Dr. Atkinson as Secretary. Dr. O'Hagan, of North Carolina, and Dr. Paul F. Eve, of Nashville, were added to the committee to visit foreign medical associations. A letter was read from Dr. Eve, regretting his forced absence from the present meeting.

*Nomenclature of Diseases.*—The chairman of the Committee on Nomenclature of Diseases submitted the following report:

In accordance with the resolutions appended to the minority report of the committee, adopted by the Association at its last meeting, one thousand extra copies of the proposed nomenclature were printed in pamphlet form, and distributed to the profession, and to the various medical journals, both at home and abroad; and such criticisms and suggestions as would represent their opinions as to its merits and fitness were invited from those receiving it. To this invitation not a single response has been made by medical journals, and but from two practitioners, the latter being such additions as in the judgment of these gentlemen would render the work more complete, but which, in the judgment of the majority, describe conditions which none but a specialist could recognize. From this statement of the results of a year's consideration of the proposed nomenclature, the conclusion may be drawn that the profession are satisfied with the work. Your committee are not willing to entertain the only other conclusion, that men of culture and practical men are indifferent upon a subject of such importance; they therefore again present the resolutions appended to the majority report and ask for their adoption:

*Resolved*, That the report of the Committee on the Nomenclature of Diseases be referred to a special committee of five members, to be appointed by the President, who shall examine it and report upon its final disposition at the present meeting of the Association.

*Resolved*, That on the favorable report of such committee, it shall be referred back to the Committee on the Nomenclature of Diseases for the preparation of an index to be published with it, in the forthcoming volume of the Transactions.

Dr. Woodward, U. S. A., spoke against the adoption of nomenclature presented by the majority, setting forth the errors and short-comings; and on his recommendation the following resolutions were adopted:

*Resolved*, That in the opinion of this Association it is inexpedient to adopt the nomenclature and classification presented by the majority of the Committee on Nomenclature at the meeting at Philadelphia

*Resolved*, That a committee of three be appointed by the President, whose duty it shall be to communicate the foregoing resolution to the proper committee of the Royal College of Physicians of London, and to negotiate for the representation of the American Medical Association in the first decennial revision of their nomenclature.

*An International Medical Congress.*—On motion of Dr. Toner, of Washington City, the following resolution was adopted:

*Resolved*, That in the opinion of this Association it would be an excellent opportunity, at the American Centennial in 1876, for an International Medical Congress to consider, and, if practicable, to adopt, a uniform classification and nomenclature of diseases, to be used by the profession throughout the world.

*From the Mediterranean.*—The President read a letter from Dr. R. R. Stover, at present at Mentone, in the Mediterranean, asking the Association to appoint a committee to compare the American and foreign winter cures. The letter was referred to the Nominating Committee.

*The Secretary's Salary.*—Dr. Kellar, of Louisville, offered the following resolution:

*Resolved*, That the sum of $500 be appropriated as an honorarium for the services of the permanent Secretary during the present year, provided such an amount remains in the treasury after paying necessary expenses.

*A National Sanitary Bureau.*—Dr. Bell, of Brooklyn, New York, offered the following resolutions:

*Resolved*, That in the judgment of this Association the establishment of a National Sanitary Bureau, with relations to the General Government similar to those of the Bureaus of Agriculture and Education, is highly desirable as a means of promoting sanitary science and the protection of the public health.

*Resolved*, That this Association request of the U. S. Educational Bureau to so extend the scope of its inquiry as to include vital diseases and mortuary statistics in relation to local, meteorological and geological influences, and to disseminate the information so collected throughout the country.

After considerable debate these resolutions were referred to the Committee on Public Hygiene and State Medicine.

*Suggestions on Medical Education.*—The hour of eleven having arrived, special business was in order, and the report of Special Committee was called, to present suggestions on the subject of medical education. Dr. Pollock, of Pittsburg, read the following report:

The committee appointed by the President, at the last meeting of the Association, to take into consideration the propriety of adopting the suggestions of the Committee on Medical Education, are fully impressed with the importance of the subject, and acknowledge the value of the suggestions offered. But we believe it wholly impracticable to carry into operation any law which does not meet the hearty approval of diverse interests connected with the teaching and practice of medicine; and while we have no doubt that this Association has grown to be a power in the profession, felt and recognized by all, yet to make that power effective, its decisions should be calm and deliberate. Therefore your committee, after due deliberation, have concluded to recommend the adoption of the conclusion of the report of the Committee on Medical Education, which is as follows: That a Congress, composed of two members from each State and Territory, and one from each recognized medical college, all to be members of this Association, be appointed (or nominated by the Nominating Committee) at this present session. That said committee, or congress, shall meet three days previous to our next annual meeting, and that said committee, or congress, shall perfect a plan for some uniform system of medical teaching, which, when adopted by the Association, shall be the only recognized method of medical teaching in the United States.

This report was referred to the Committee on Nominations; then, after long debate, brought back and laid on the table.

*The U. S. Marine Hospital Service.*—The following amendment to the Constitution of the American Medical Association, as to its relations with the U. S. Marine Hospital Service, proposed by Dr. Hartshorne, of Philadelphia, at the last annual session of the Association, was brought up and referred to the next year's meeting. Its purpose is to place the United States Marine Hospital Service in the same relative position in the American Medical Association as the Medical Departments of the United States Army and Navy.

*The Meeting at Detroit.*—It was resolved that the next annual meeting, to be held at Detroit; commence on the first Tuesday in June, as it will be too cold in that locality to assemble together early in May, as usual.

*Alcoholic Stimulants.*—Some temperance man from the rural district wanted the Association to emphatically declare against the free use of alcohol in prescriptions, etc. It was stated by the chair that a caution against the too free use of alcohol had been declared last year, and the extra teetotaler, with his motion, was ruled out of order.

*Election of Officers.*—The following amendment to the Constitution was proposed and laid over, under the rule, for one year:

*Resolved*, That article 12, of the Constitution, be amended as follows: Strike out the second clause of first paragraph and insert "They shall be nominated by the Judicial Council and shall be elected by vote on a general ticket."

The Committee on Nominations, after a prolonged absence, returned and reported as follows through the chairman, Dr. Johnson, of St. Louis:

*Chairmen and Secretaries of Sections for* 1874.—1. Practice of Medicine, Materia Medica and Physiology—Dr. N. S. Davis, of Chicago, Chairman; and Dr. Frothingham, of Ann Arbor, Michigan, Secretary.

2. Obstetrics and Diseases of Women and Children—Dr. Theo. Parvin, of Indianapolis, Chairman; and Dr. Montrose A. Pallen, of St. Louis, Secretary.

3. Surgery and Anatomy—Dr. S. D. Gross, of Philadelphia, Chairman; and Dr. Alonzo Garcilon, of Maine, Secretary.

4. Medical Jurisprudence, Chemistry and Psychology—Dr. A. N. Talley, of South Carolina, Chairman; and Dr. E. L. Howard, of Maryland, Secretary.

5. State and Public Hygiene—Dr. A. N. Bell, of Brooklyn, Chairman; and Dr. A. B. Stuart, of Winona, Minnesota, Secretary.

*Resolved*, That the Secretary of the Association be authorized to fill up all vacancies in the Committee from the States and Territories.

Also, that Dr. A. N. Bell be President, and Dr. A. B. Stuart, as Secretary.

*Committee on Necrology.*—Chairman, Dr. A. Sager, Ann Arbor, Michigan, and committee to remain as last year, excepting Dr. Alonzo Garcilon, of Maine, in place of Dr. D. McReese, deceased.

*Judicial Committee.*—Three years—Drs. Brodie, Mich.; N. S. Davis, Ill.; E. L. Howard, Md.; Wm. O'Baldwin, Ala.; Dean, N. Y.; J. P. Logan, Ga.

Two years—Drs. L. S. Joynes, Va.; R. N. Todd, Ind.; Askew, Del.; J. E. Morgan, D. C.; Daniel Lillie, N. J.; S. N. Benham, Pa.; Dunlap, Conn.

One year—Drs. Bartlett, Wis.; Powell, Ill.; Gale, Ky.; Moses, Mo.; Hughs, Iowa; Bemnis, La.; Cheever, Mass.

*Special Committee.*—Dr. H. R. Storer, Boston, Mass., Chairman of committee to report on American as compared with foreign winter cures.

The report was adopted by the Association *in toto*.

(It was my intention, Mr. Editor, to have kept up a running commentary all the way through, especially upon the section work, but I soon found that it would encroach too much on your space. I may say only in conclusion that the Association fulfilled the expectations of every one; that is, no one expected it would do anything, and it did just simply—nothing. And thus endeth the 24th session of the great American Mudfog Association for the Advancement of Everything and the Accomplishment of Nothing.)

John Smith, M.D.

—*The Clinic.*

